# Investigation of Low-Temperature Molten Oxide Electrolysis of a Mixture of Hematite and Zinc Oxide

**DOI:** 10.3390/ma18174116

**Published:** 2025-09-02

**Authors:** Joongseok Kim, In-Ho Jung, Jungshin Kang, Kyung-Woo Yi

**Affiliations:** 1 Department of Materials Science and Engineering, Seoul National University, 1 Gwanak-ro, Gwanak-gu, Seoul 08826, Republic of Korea; joongseok10@snu.ac.kr (J.K.); in-ho.jung@snu.ac.kr (I.-H.J.); 2 Research Institute of Advanced Materials, Seoul National University, 1 Gwanak-ro, Gwanak-gu, Seoul 08826, Republic of Korea; 3 Department of Energy Resources Engineering, Seoul National University, 1 Gwanak-ro, Gwanak-gu, Seoul 08826, Republic of Korea; 4 Research Institute of Energy and Resources, Seoul National University, 1 Gwanak-ro, Gwanak-gu, Seoul 08826, Republic of Korea

**Keywords:** high-purity iron, high-purity zinc, secondary resources, molten oxide electrolysis, vacuum distillation

## Abstract

To develop a CO_2_-free process for recovering Fe and Zn metals from electric arc furnace (EAF) dust, this study investigated the molten oxide electrolysis of various Fe_2_O_3_–ZnO mixtures in a B_2_O_3_–Na_2_O electrolyte. Electrolysis was conducted using an Fe cathode and Pt anode at 1173 K by applying cell voltages that were determined based on thermodynamic calculations and cyclic voltammetry measurements. When electrolysis was conducted at a cell voltage of 1.1 V, the selective reduction of Fe oxide to Fe metal was observed without ZnO reduction. However, when 1.6 V was applied, the co-reduction of Fe oxide and ZnO to the Fe–Zn alloy was observed. In the vacuum distillation of the Fe–Zn alloy at 1000–1200 K, Zn metal with a purity of ≥99.996% was obtained with a recovery efficiency of ≥99.9%, under certain conditions. This study demonstrates the feasibility of recovering Fe and Zn from EAF dust using low-temperature molten oxide electrolysis and subsequent vacuum distillation.

## 1. Introduction

The production of iron (Fe) and steel, which are fundamental commodities, has rapidly increased since the 20th century owing to the development of the global economy. For example, the global pig Fe and steel production in 2023 exceeded 1.3 and 1.8 billion tons, respectively [1,2]. Consequently, the generation of by-products, such as dust, has also increased. Although most of the produced dust can be directly recycled as raw material for blast furnaces, electric arc furnace (EAF) dust is not directly recycled because of its high zinc (Zn) concentration [3]. EAF dust is currently recycled using the Waelz kiln or the rotary hearth furnace (RHF) process. In these processes, Zn (*g*) and Fe (*s*) are recovered by reducing EAF dust using carbon (C) at temperatures above 1273 K, resulting in carbon monoxide (CO) and/or carbon dioxide (CO_2_) gas emissions. Considering the environmental burden of these processes, developing a CO_2_-free method for recycling EAF dust is essential in the near future.

Various technologies have been developed in the Fe and steel industry to reduce CO_2_ emissions. An important approach involves changing the reducing agent from coke to hydrogen (H_2_) gas or electrons [4]. Although the commercialization of molten oxide electrolysis is currently under investigation, it has been reported to offer advantages in terms of energy consumption and CO_2_ emissions [5,6,7]. Moreover, because CO_2_-free Fe production with H_2_ requires green H_2_ generated by water electrolysis, directly using electrons as reducing agents for Fe production can simplify the process.

Numerous studies have investigated the electrolysis of Fe oxides, as shown in Table 1. Electrolysis using molten oxides, salts, carbonates, and hydroxides has been investigated using various supporting electrolytes. Among the various electrolysis methods, molten oxide electrolysis is promising owing to the high solubility of oxide feedstocks and the direct use of oxide feeds without pre-treatment, such as sintering or pelletizing. Several studies have reported the results of molten oxide electrolysis at high temperatures such as 1700 K, as shown in Table 1. However, operation at such high temperatures results in higher energy consumption compared to other electrolytic processes that use molten salts, carbonates, or hydroxides.

To decrease the energy consumption of molten oxide electrolysis, the low-temperature molten oxide electrolysis of hematite (Fe_2_O_3_) using a boron oxide (B_2_O_3_)–sodium oxide (Na_2_O) electrolyte was investigated [14]. When conducted at 1273 K by applying 1.4–2 V, Fe metal was obtained with a current efficiency of 32.3–54.7%. Use of the B_2_O_3_–Na_2_O electrolyte retained the advantages of the molten oxide electrolysis process, such as the sufficient solubility of Fe_2_O_3_ even at moderate operating temperatures.

However, investigations into electrolytic methods using EAF dust to recover Fe and Zn have rarely been reported, as shown in Table 1. Liu et al. performed electrolysis of ZnFe_2_O_4_ generated during Zn metallurgy instead of EAF dust in sodium chloride (NaCl)–calcium chloride (CaCl_2_) molten salt at 1073 K [22]. ZnFe_2_O_4_ underwent stepwise reduction, and Fe was reduced before Zn. Although this process can be used to recycle EAF dust, treating chloride salts is challenging because the electrolysis environment tends to be corrosive owing to their hygroscopicity.

**Figure 1:**
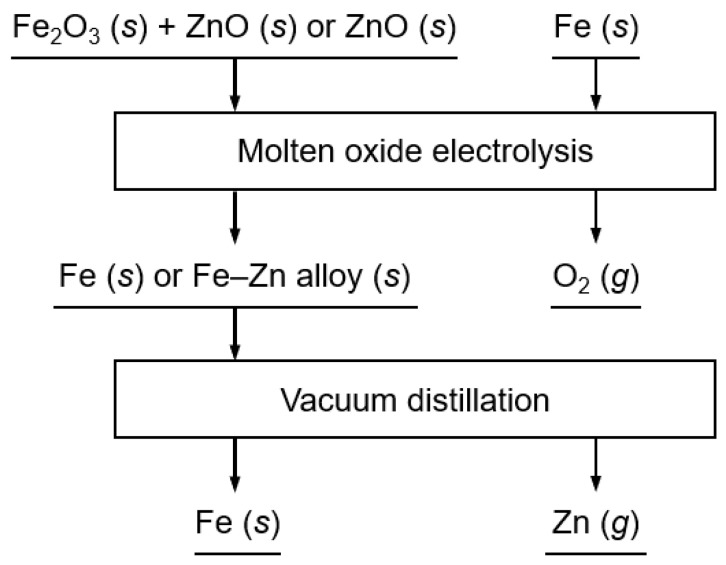
Flowchart of the Fe and Zn production process presented in this study.

Low-temperature molten oxide electrolysis is an alternative method for recovering Fe and Zn from EAF dust. Molten oxides offer a safer and more stable electrolysis environment than chloride-based molten salt systems. In addition, Zn and Fe can be recovered from EAF dust without emitting CO_2_ gas by using an inert anode. Therefore, to develop a novel and environmentally friendly method for recycling EAF dust, we fundamentally investigated the electrochemical behavior of Fe and Zn oxides and their electrolysis at 1173 K using low-temperature molten oxide electrolysis, as shown in Figure 1.

## 2. Materials and Methods

### 2.1. Cyclic Voltammetry (CV) of Fe_2_O_3_ and ZnO in B_2_O_3_–Na_2_O Molten Oxide

CV measurements were conducted to investigate the electrochemical behavior of Fe_2_O_3_ and ZnO in B_2_O_3_–Na_2_O molten oxide at 1173 K. Prior to the experiments, Na_2_O (purity: >97.5%; Thermo Fisher Scientific Chemicals, Inc., Ward Hill, MA, USA) was dried in a vacuum oven (Model: VOS-602 SD, EYELA, Tokyo, Japan) at 453 K for more than 24 h and then stored in a glove box (Model: MB-200MOD, MBRAUN, Garching, Germany) to prevent hydration by atmospheric moisture.

An oxide mixture of 71 mass% B_2_O_3_ (purity: 99.98%; Thermo Fisher Scientific Chemicals, Inc., Ward Hill, MA, USA), 26 mass% Na_2_O, and 3 mass% Fe_2_O_3_ (purity: 99.9%; Thermo Fisher Scientific Chemicals, Inc., Ward Hill, MA, USA) and a mixture of 71 mass% B_2_O_3_, 26 mass% Na_2_O, 1.5 mass% Fe_2_O_3_, and 1.5 mass% ZnO (purity: 99.99%; Thermo Fisher Scientific Chemicals, Inc., Ward Hill, MA, USA) were prepared at room temperature. The oxide mixtures were placed in an alumina (Al_2_O_3_) crucible (O.D. = 56 mm, thickness (*t*) = 3.5 mm).

**Figure 2:**
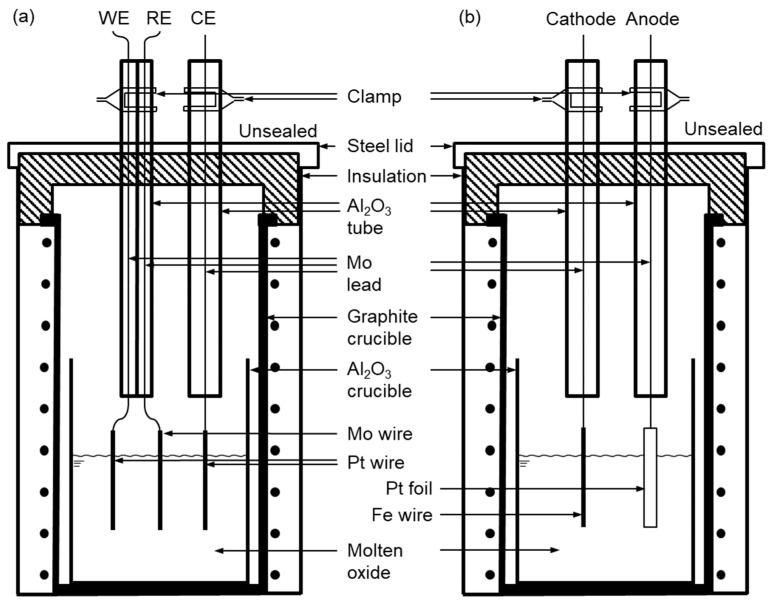
Schematic of experimental apparatus used for (**a**) cyclic voltammetry measurement and (**b**) electrolysis in this study.

Figure 2a shows a schematic of the electrolytic cell used for the CV measurements (see Figure S1 in the Supplementary Materials for a photograph of the apparatus). An Al_2_O_3_ crucible containing the oxide mixture was placed in an electric furnace and the temperature was increased to 1173 K for the CV measurements.

Platinum (Pt) wire (purity: 99.95%; diameter (*ϕ*) = 0.5 mm; Thermo Fisher Scientific Chemicals, Inc., Ward Hill, MA, USA) was used as both the working and counter electrodes whereas molybdenum (Mo) wire (purity: 99.95%; *ϕ* = 0.5 mm; Thermo Fisher Scientific Chemicals, Inc., Ward Hill, MA, USA) was used as the quasi-reference electrode. In addition, an identical Mo wire was used as the potential lead for all electrodes. The electrodes and Mo potential lead were hand-polished prior to the experiment. After connecting the electrode and potential lead, the potential leads of the working and quasi-reference electrodes were diagonally inserted into two holes of an Al_2_O_3_ tube (O.D. = 8.5 mm) with four holes (*ϕ* = 1.8 mm). The potential lead of the counter electrode was inserted into a hole in an identical Al_2_O_3_ tube. The end of the potential lead connected to the potentiostat was bent and secured using Teflon tape to prevent electrode movement. Subsequently, the prepared Al_2_O_3_ tubes were inserted into the furnace through the two holes in the lid and fixed with clamps. The prepared electrodes were immersed in molten oxide, and CV measurements were conducted using a potentiostat (Model: SP-150e, booster: VMP3B, 2 A–20 V, Biologic Science Instruments, Seyssinet-Pariset, France).

### 2.2. Molten Oxide Electrolysis of Fe_2_O_3_ and ZnO Using the Fe Cathode

Before the experiments, Na_2_O was dried in a vacuum oven at 453 K for 24 h and then stored in a glove box. Subsequently, 3.0 g of the Fe_2_O_3_–ZnO mixture with a predetermined composition as listed in Table 2 was mixed with 97.0 g of an oxide mixture containing B_2_O_3_ and Na_2_O at 73 and 27 mass%, respectively. The prepared oxide mixture was placed in an Al_2_O_3_ crucible and then placed in an electric furnace. The temperature was increased to 1173 K and maintained for 3 h before electrolysis.

Figure 2b shows a schematic of the experimental apparatus used for molten oxide electrolysis. Fe wire (purity: 99.99%; *ϕ* = 1 mm; Thermo Fisher Scientific Chemicals, Inc., Ward Hill, MA, USA) was used as the cathode, and Pt foil (purity: 99.9%; length (*l*) = 20 mm, width (*w*) = 3 mm, *t* = 0.127 mm; Thermo Fisher Scientific Chemicals, Inc., Ward Hill, MA, USA) was used as the anode. A Mo wire was used as the potential lead to connect the electrode and potentiostat. The electrodes and Mo potential lead were hand-polished before use. Each potential lead assembled with an electrode was inserted into a separate Al_2_O_3_ tube. The end of the potential lead connected to the potentiostat was bent and secured using Teflon tape. Subsequently, the prepared Al_2_O_3_ tubes were inserted into the furnace through the two holes in the lid and fixed with clamps. The prepared electrodes were immersed in the molten oxide and chronoamperometry was performed in the range of 1.1–1.6 V at 1173 K. Following electrolysis completion, the electrodes were removed from the molten oxide. Subsequently, the temperature was decreased to room temperature, and the cathode was retrieved for analysis.

### 2.3. Vacuum Distillation of the Fe–Zn Alloys

**Figure 3:**
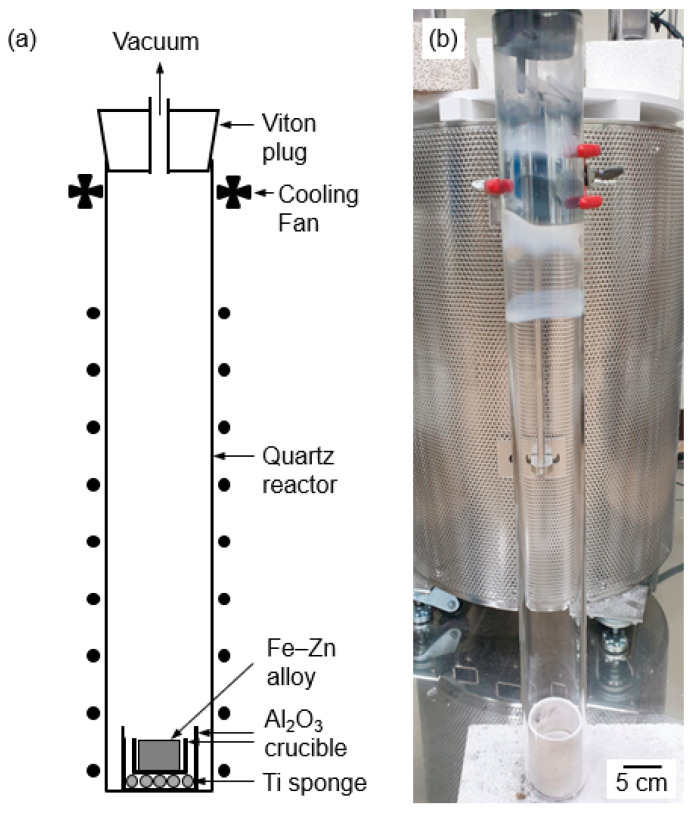
(**a**) Schematic and (**b**) photograph of the experimental apparatus for vacuum distillation.

Figure 3 shows a schematic and photograph of the experimental apparatus used for vacuum distillation. The Fe–27.1 mass% Zn alloy (*l* = 10 mm, *w* = 10 mm, *t* = 10 mm; RND KOREA Corp., Gwanmyeong, Republic of Korea) was placed in a small Al_2_O_3_ crucible (O.D. = 40 mm, *t* = 2 mm) that was then placed in another Al_2_O_3_ crucible (O.D. = 50 mm, *t* = 2 mm). A titanium (Ti) sponge was packed at the bottom to absorb residual oxygen in the atmosphere at elevated temperatures. Subsequently, the assembly was positioned at the bottom of a quartz reactor (O.D. = 61 mm, *t* = 3 mm, height (*h*) = 650 mm).

A Viton plug equipped with Pyrex tubes as the inlet and outlet was plugged into the top of the reactor, which was then evacuated and refilled twice with argon (Ar) gas (purity: 99.999%) to control the atmosphere. After the final filling with Ar gas, the reactor was continuously evacuated using a rotary pump until the end of the experiment.

The reactor was placed in a furnace preheated to 1200 K for the vacuum distillation of Fe–Zn alloy for 1–12 h. After vacuum distillation, the reactor was immediately removed from the furnace and cooled to room temperature. The residue at the bottom of the crucible and the deposit on the inner wall of the reactor were recovered for analysis.

### 2.4. Analysis

The microstructures and compositions of the samples were analyzed using field-emission scanning electron microscopy (FE-SEM: GeminiSEM 560, Carl Zeiss AG, Jena, Germany) equipped with energy-dispersive X-ray spectroscopy (EDS: Ultim Max 100, Oxford Instruments, High Wycombe, UK). The crystalline phases of samples were identified using X-ray diffractometer (XRD: SmartLab, Rigaku Corporation, Tokyo, Japan, Cu-Kα radiation). The compositions of the samples were analyzed using inductively coupled plasma optical emission spectroscopy (ICP-OES: 5800 ICP-OES, Agilent Technologies, Santa Clara, CA, USA).

## 3. Electrolysis Mechanism of Fe_2_O_3_ and ZnO in Molten B_2_O_3_–Na_2_O

In this study, to produce metallic Fe or Fe–Zn alloy from Fe_2_O_3_ and ZnO, electrolysis in molten B_2_O_3_–Na_2_O using an Fe cathode and a Pt anode was investigated. The FactSage thermodynamic software (FactSage 8.3 version; www.factsage.com) was employed to understand the electrolysis mechanism of Fe_2_O_3_ and ZnO. In particular, the FTOxid database containing optimized data for the Fe_2_O_3_–B_2_O_3_–Na_2_O and ZnO–B_2_O_3_–Na_2_O systems, in which thermodynamic assessments are based on the modified quasi-model [29,30], was used to calculate activities of molten oxides. In addition, the FSStel database for the Fe–Zn alloy was utilized in the FactSage calculations.

**Figure 4:**
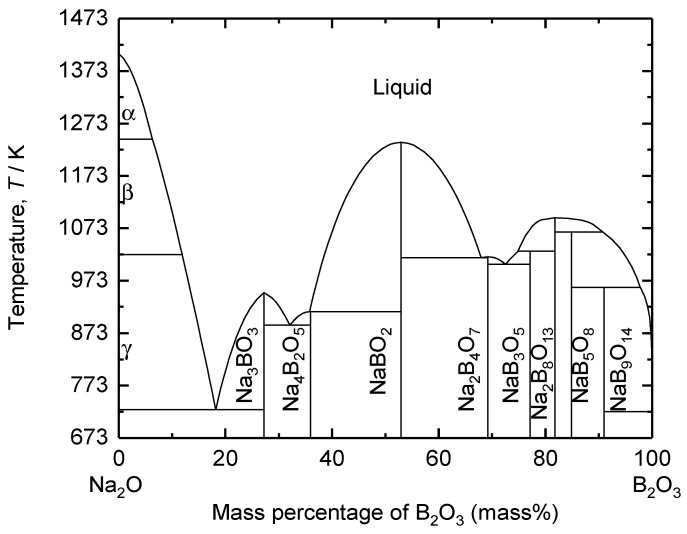
Calculated binary phase diagram of the B_2_O_3_–Na_2_O system.

The composition of the supporting electrolyte was determined based on the binary phase diagram of B_2_O_3_–Na_2_O shown in Figure 4. The eutectic compositions of this system were calculated to be at 73 mass%, 32 mass%, and 18 mass% B_2_O_3_. Na_2_O is a network modifier that breaks down the B_2_O_3_ network structure in a borate melt, leading to an increase in electrical conductivity [31,32,33]. However, Kim et al. demonstrated that increasing the amount of basic oxide species increases the basicity of the melt, thereby leading to the corrosion of an iridium (Ir)-based inert anode [34]. Therefore, 73 mass% B_2_O_3_–Na_2_O was selected as the supporting electrolyte in this study.

As shown in Figure 4, the eutectic temperature of 73 mass% B_2_O_3_–Na_2_O is 1004 K. Considering the composition as well as the temperature fluctuations during the scaled-up process in the future, the electrolysis temperature of 1173 K was selected for the stable electrolysis operation.

Fe and Zn in EAF dust typically exist in the form of zinc ferrite (ZnFe_2_O_4_). However, when ZnFe_2_O_4_ is dissolved in the B_2_O_3_–Na_2_O melt, Fe and Zn are present as dissolved species. Therefore, instead of using ZnFe_2_O_4_, the mixture of Fe_2_O_3_ and ZnO was used as the feedstock to assess the feasibility of utilizing actual EAF dust for molten oxide electrolysis.

**Figure 5:**
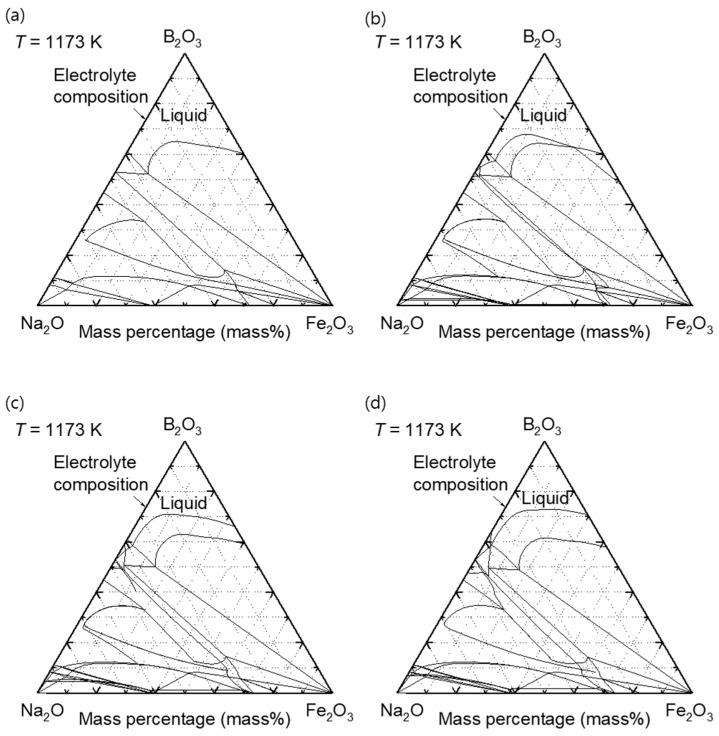
Calculated phase diagrams of the B_2_O_3_–Na_2_O–Fe_2_O_3_–ZnO system at 1173 K with constant ZnO contents of (**a**) 0 mass%, (**b**) 1 mass%, (**c**) 2 mass%, and (**d**) 3 mass%.

The solubilities of Fe_2_O_3_ and ZnO in molten B_2_O_3_–Na_2_O at 1173 K were calculated using the FactSage software and were found to be 11.7 mass% and 4.4 mass%, respectively. Consequently, the maximum concentration of ZnO added to 73 mass% B_2_O_3_–Na_2_O was 3 mass% in this study. The feedstock compositions used in this study are listed in Table 2. Figure 5 shows the iso-composition plane of the quaternary phase diagram of the B_2_O_3_–Na_2_O–Fe_2_O_3_–ZnO system at 1173 K with 0 mass%, 1 mass%, 2 mass%, and 3 mass% ZnO. According to the calculated phase diagrams presented in Figure 5, all oxide mixtures were in a fully liquid state.

Table 3 shows the theoretical standard decomposition voltages of the selected oxides at 1173–1373 K. Na_2_O and B_2_O_3_ are more stable than the other oxides, owing to their higher decomposition voltages. The reductions of Fe_2_O_3_ and ZnO proceed in the following order: Fe_2_O_3_ to wüstite (FeO), FeO to metallic Fe, and ZnO to liquid Zn. However, because the actual electrolytic process does not proceed under the standard state conditions, it is necessary to consider the activities of the oxides in the electrolyte melt and the metals at the Fe cathode to accurately estimate the decomposition voltages of all species involved during electrolysis at 1173 K. Thus, FactSage thermodynamic calculations were performed.

**Figure 6:**
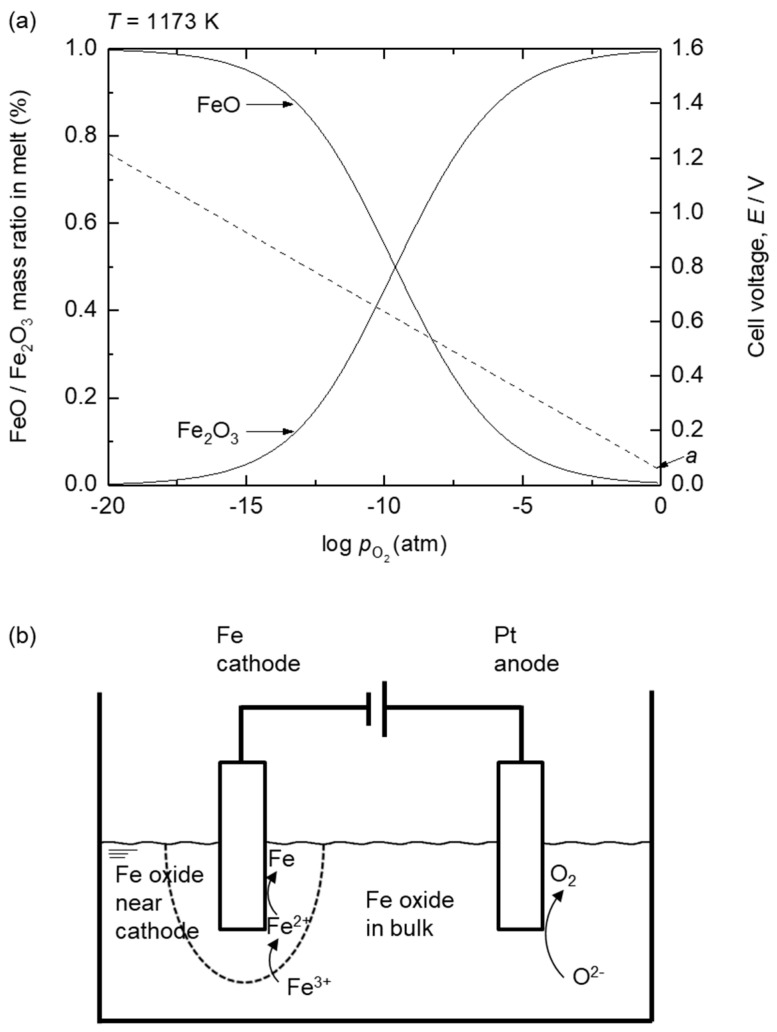
(**a**) Variations in Fe_2_O_3_ and FeO ratio in the melt and the decomposition voltages for the reduction of Fe_2_O_3_ to FeO as a function of $p_{O_{2}}$ at 1173 K, calculated from the FactSage database: electrolyte composition of 73 mass% B_2_O_3_–Na_2_O. (**b**) Illustration of the reactions during the electrolysis of Fe oxide in oxide melt.

In the case of Fe, trivalent (Fe^3+^) and divalent (Fe^2+^) Fe ions are present in the oxide melt. To calculate the decomposition voltages of Fe_2_O_3_ and FeO, their activities in the melt must be considered. Figure 6a shows the variations in Fe_2_O_3_ and FeO in the 73 mass% B_2_O_3_–Na_2_O electrolyte at 1173 K as a function of oxygen partial pressure ($p_{O_{2}}$). Using the Nernst equation, the cell voltage required for the reduction of Fe_2_O_3_ to FeO under each $p_{O_{2}}$ condition was calculated and plotted in Figure 6a. As shown in Figure 6a, Fe^3+^ and Fe^2+^ are dominant at high $p_{O_{2}}$ and low $p_{O_{2}}$, respectively. During electrolysis, reducing conditions are formed near the cathode, corresponding to a low $p_{O_{2}}$, whereas oxidizing conditions are formed near the anode via O_2_ gas evolution, corresponding to a high $p_{O_{2}}$. Therefore, as electrolysis proceeds, Fe^2+^ primarily exists near the cathode, followed by the reduction of Fe^3+^ to Fe^2+^, whereas Fe^3+^ will primarily exist near the anode, as shown in Figure 6b.

**Figure 7:**
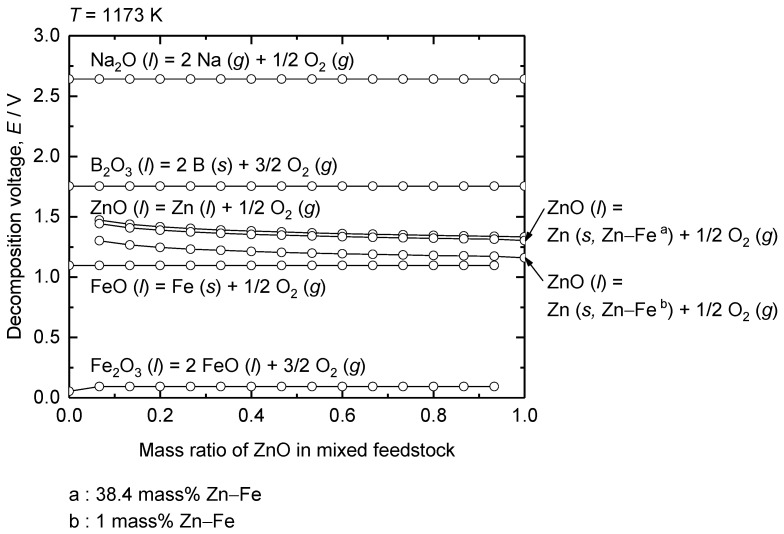
Decomposition voltages of selected oxides as a function of ZnO concentration in mixed feedstock.

Figure 7 shows the estimated decomposition voltages of Fe_2_O_3_, FeO, ZnO, B_2_O_3_, and Na_2_O using the Nernst equation, considering their activities under the conditions of this study at 1173 K as a function of the ZnO ratio in the Fe_2_O_3_–ZnO mixed feed. Before electrolysis, Fe_2_O_3_ is dominant over FeO because the Fe_2_O_3_–ZnO–B_2_O_3_–Na_2_O system is in equilibrium with air ($p_{O_{2}}$ = 0.21 atm) at 1173 K. When the onset of the electrolysis near the cathode is considered, the estimated decomposition voltage for reduction of Fe_2_O_3_ to FeO decreases from 0.74 V under standard state conditions to approximately 0.05–0.09 V owing to the significantly low *a*_FeO_ in the melt.

The decomposition voltage for the reduction of FeO to Fe was calculated by considering the activity of Fe as unity owing to the formation of pure metallic Fe (*s*). Accordingly, the decomposition voltage for the reduction of FeO to Fe is governed by *a*_FeO_, which is directly affected by the FeO concentration near the cathode. Because the decomposition voltage for the reduction of Fe_2_O_3_ at the onset of electrolysis is considerably low as shown in Figure 7, Fe_2_O_3_ will be reduced to FeO before the reduction of FeO to metallic Fe. This results in the accumulation of FeO near the cathode, leading to a gradual increase in its concentration, until it is reduced to metallic Fe.

The equilibrium $p_{O_{2}}$ for the Fe (*s*)/FeO (*l*, in melt) eq. under the experimental conditions is 10^−18^ atm at 1173 K. Therefore, the activities of the existing state of Fe oxides such as *a*_FeO_ and *a*_Fe2O3_ before the reduction of FeO to metallic Fe are determined by the FeO (*l*, in melt)/Fe_2_O_3_ (*l*, in melt) eq. under $p_{O_{2}}$ = 10^−18^ atm. The resulting decomposition voltage estimated using *a*_FeO_ was approximately 1.09 V, which is slightly higher than that calculated under the standard state of 0.98 V. This increase is attributed to the lower *a*_FeO_ content in the melt compared with that in the standard state.

**Figure 8:**
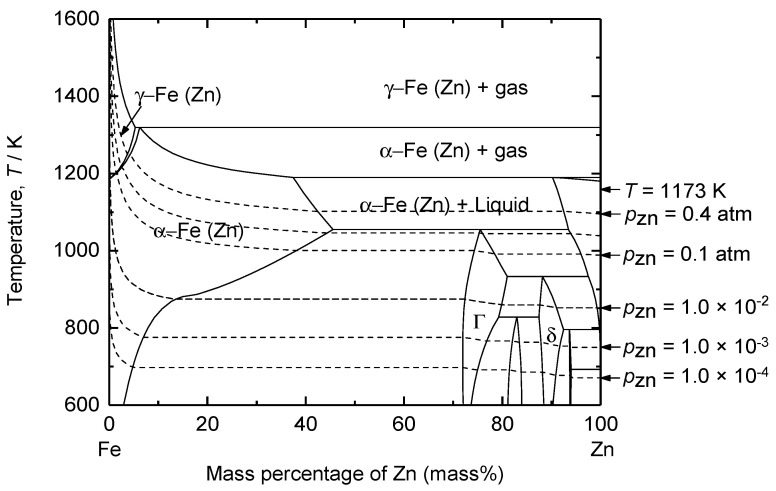
Calculated binary phase diagram of the Fe–Zn system at 1 atm total pressure. Dotted lines indicate the isobaric vapor pressure of Zn (*g*).

The decomposition voltage for the reduction of ZnO was calculated by considering the activities of ZnO near the cathode and Zn in the Fe–Zn alloy. Figure 8 shows that the solubility of Zn in Fe is 38.4 mass% at 1173 K. Consequently, the activities of Zn in the 1 mass% Zn–Fe and 38.4 mass% Zn–Fe solid solutions were considered to estimate the decomposition voltage of ZnO. The *a*_Zn_ of the 1 mass% Zn–Fe solid solution indicates the onset of ZnO reduction into the Fe cathode, whereas that of the 38.4 mass% Zn–Fe solid solution indicates the saturation of Zn in the Fe cathode by ZnO reduction. The estimated decomposition voltages were 1.21 V and 1.34 V for 1 mass% and 38.4 mass% Zn–Fe solid solutions, respectively.

For the supporting electrolytes, the calculation results indicate that both B_2_O_3_ and Na_2_O decompose at higher voltages than Fe oxides and ZnO at 1173 K. The estimated decomposition voltage for B_2_O_3_ (*l*, in melt) was 1.75 V, which is slightly higher than that of B_2_O_3_ (*l*) under standard state conditions (1.70 V). The estimated decomposition voltage of Na_2_O (*l*, in melt) was 2.64 V, which is significantly higher than that of Na_2_O (*s*) under standard state conditions (1.33 V). This increase is attributed to a substantial decrease in the activity of Na_2_O in the melt. Notably, Na_2_O exhibits considerably low activity in acidic oxide systems because it is strongly stabilized in melt [35,36,37].

These results indicate that the reduction of Fe and Zn occurs prior to the decomposition of the electrolyte components. In addition, the selective reduction of FeO from the mixture of Fe_2_O_3_ and ZnO is feasible through molten oxide electrolysis at 1173 K by using the difference in their decomposition voltages, as shown in Figure 7. Furthermore, when the cell voltage above the decomposition voltage determined by the ZnO (*l*, in melt)/1 mass% Zn–Fe (*s*) eq. is applied during the molten oxide electrolysis of the mixture of Fe_2_O_3_ and ZnO at 1173 K, an Fe–Zn solid solution will be obtained.

## 4. Results and Discussion

### 4.1. CV Measurements of Fe_2_O_3_ and the Mixture of Fe_2_O_3_ and ZnO in the Molten Oxide

**Figure 9:**
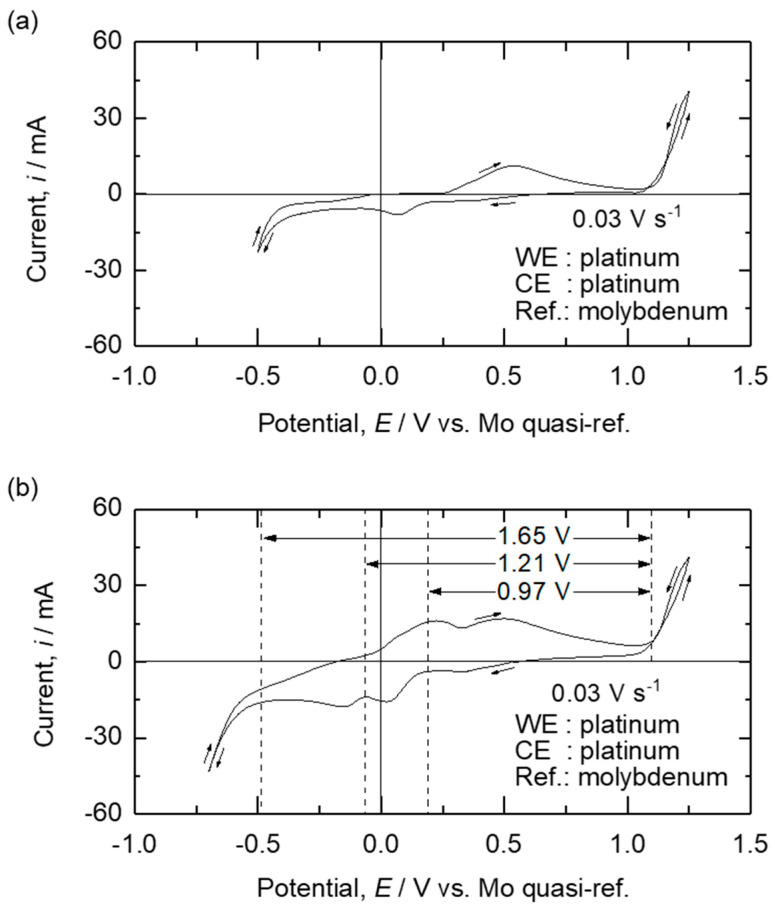
Results of the CV measurements of (**a**) B_2_O_3_–Na_2_O–Fe_2_O_3_ and (**b**) B_2_O_3_–Na_2_O–Fe_2_O_3_–ZnO molten oxides at 1173 K with a scan rate of 0.03 V·s^−1^.

To investigate the electrochemical behaviors of the Fe and Zn oxides in the B_2_O_3_–Na_2_O molten oxides at 1173 K, CV measurements were conducted using Mo as the quasi-reference electrode and Pt as both the working and counter electrodes. Figure 9 shows the results of the CV measurements conducted on the B_2_O_3_–Na_2_O–Fe_2_O_3_ molten oxide before and after the addition of ZnO.

As shown in Figure 9a,b, a sharp increase in anodic current was observed at 1.15 V (vs. Mo quasi-reference electrode). This increase is attributed to the oxidation of O^2−^ ions to O_2_ gas, as shown in Equation (1). In addition, a sharp increase in cathodic current was observed at −0.5 V (vs. Mo quasi-reference electrode). As shown in Figure 7, the estimated decomposition voltages of B_2_O_3_ (*l*, in melt) and Na_2_O (*l*, in melt) were 1.75 V and 2.64 V, respectively. Therefore, the increase in the cathodic current from −0.5 V (vs. Mo quasi-reference electrode) is attributed to the reduction of B^3+^, as shown in Equation (2).

2 O^2−^ (in melt) = O_2_ (*g*) + 4 e^−^ (1)

B^3+^ (in melt) + 3 e^−^ = B (*s*) (2)

As shown in Table 3, the decomposition voltages of Na_2_O and B_2_O_3_ are 1.33 V and 1.70 V at 1173 K under the standard state, respectively. The decomposition voltage of Na_2_O is lower than that of B_2_O_3_ under the standard state. It is worth noting that Na^+^/Na (*l*) eq. was not observed in Figure 9. This shows that the results of the CV measurements match the estimated decomposition voltages of B_2_O_3_ (*l*, in melt) by the FactSage software for the Fe_2_O_3_–B_2_O_3_–Na_2_O and ZnO–B_2_O_3_–Na_2_O systems. Therefore, the utilization of the optimized thermodynamic database is valid for estimating the electrochemical behavior of the molten oxide electrolysis of the B_2_O_3_–Na_2_O–Fe_2_O_3_–ZnO system at 1173 K.

Meanwhile, as shown in Figure 9a,b, when the potential of the working electrode decreased from 1.15 V (vs. Mo quasi-reference electrode) to 0.4 V (vs. Mo quasi-reference electrode), a gradual increase in the cathodic current was observed. Because the estimated potential for O^2−^/O_2_ (*g*) eq. was 1.15 V (vs. Mo quasi-reference electrode), the value of $p_{O_{2}}$ is expected to be approximately 1 atm at 1.15 V (vs. Mo quasi-reference electrode). This value corresponds to point *a* in Figure 6 where Fe_2_O_3_ is dominant. In this case, the estimated decomposition voltage of Fe_2_O_3_ is 0.05 V. Therefore, the gradual increase in the cathodic current from 1.15 V (vs. Mo quasi-reference electrode) is attributed to the reduction of Fe^3+^ to Fe^2+^, as shown in Equation (3).

Fe^3+^ (in melt) + e^−^ = Fe^2+^ (in melt) (3)

In addition, an increase in cathodic current was observed at 0.18 V (vs. Mo quasi-reference electrode), as shown in Figure 9a,b. The span of the potential between O_2_ gas evolution and 0.18 V is 0.97 V. As the cathodic potential was swept in the negative direction from the potential of Fe^3+^/Fe^2+^ eq. at 1.15 V (vs. Mo quasi-reference electrode), the concentration of FeO near the working electrode continuously increased. In addition, Figure 7 shows that the estimated decomposition voltage of FeO (*l*, in melt) was 1.10 V. Therefore, the reduction of Fe^2+^ to Fe (*s*), as shown in Equation (4) occurred at 0.18 V (vs. Mo quasi-reference electrode).

Fe^2+^ (in melt) + 2 e^−^ = Fe (*s*) (4)

As the cathodic potential was further swept in the negative direction, an increase in the cathodic current was observed at −0.06 V (vs. Mo quasi-reference electrode) in Figure 9b. This increase was not observed when only Fe_2_O_3_ was used as the feedstock. Therefore, the cathodic current at −0.06 V (vs. Mo quasi-reference electrode) is attributed to the reduction of ZnO. The span of the potential between O_2_ gas evolution and −0.06 V is 1.21 V. As shown in Figure 7, the estimated decomposition voltage of ZnO (*l*, in melt) to Zn (*s*, in 1 mass% Zn–Fe) was 1.22 V. Therefore, the reduction of Zn^2+^ to Zn (*s*, in 1 mass% Zn–Fe), as shown in Equation (5), occurred at −0.06 V (vs. Mo quasi-reference electrode). Therefore, the reduction potentials of the dissolved oxide species were determined via CV measurements. The reduction potentials obtained during repeated CV measurements yielded identical values, validating the repeatability and the stability of the Mo quasi-reference electrode.

Zn^2+^ (in melt) + 2 e^−^ + Fe (*s*) = 1 mass% Zn–Fe (*s*) (5)

### 4.2. Electrolysis of ZnO and the Mixture of Fe_2_O_3_ and ZnO in the Molten Oxide

#### 4.2.1. Selective Reduction of the Fe Oxide from B_2_O_3_–Na_2_O–Fe_2_O_3_–ZnO Melt

To evaluate the feasibility of selectively reducing Fe oxide from a mixed feedstock containing Fe_2_O_3_ and ZnO, electrolysis was conducted by applying a cell voltage of 1.1 V for 1 h using an Fe cathode and a Pt anode in the B_2_O_3_–Na_2_O–Fe_2_O_3_–ZnO molten oxides at 1173 K. The applied cell voltage was determined by considering the results of the CV measurements shown in Figure 9, which indicated that Fe oxide can be reduced at 0.97 V, whereas the reduction of ZnO required a higher cell voltage of 1.21 V. To investigate the influence of the composition variation of Fe_2_O_3_ and ZnO in the EAF dust, feedstocks with different ratios of Fe_2_O_3_ to ZnO, as listed in Table 2, were employed.

**Figure 10:**
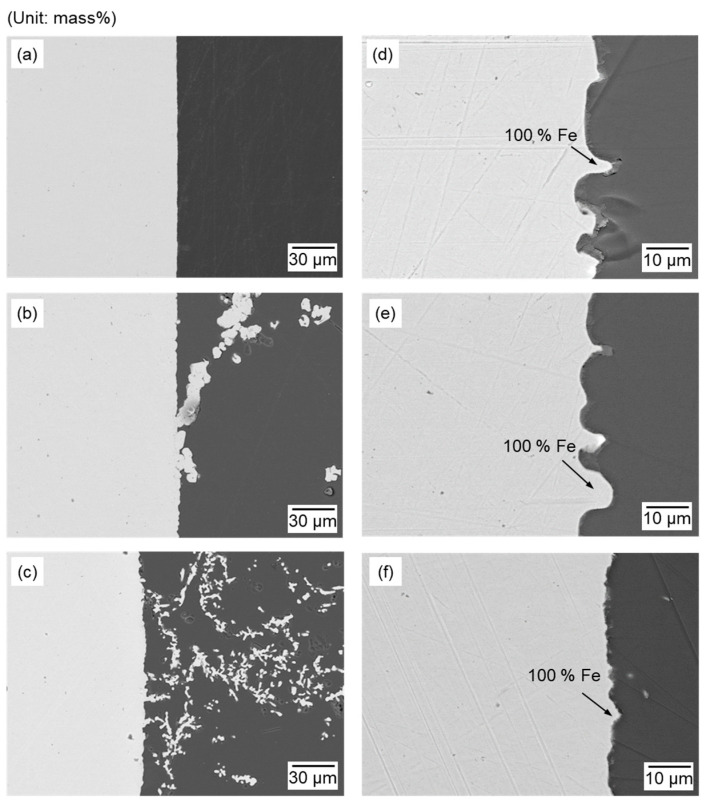
SEM-EDS analysis results of the cathode surface (**a**) before and after electrolysis at 1173 K for 1 h under the following conditions: (**b**) 1.1 V, 0.75 g of Fe_2_O_3_ + 2.25 g of ZnO (Exp. no. 1-3); (**c**) 1.6 V, 0.75 g of Fe_2_O_3_ + 2.25 g of ZnO (Exp. no. 2-3); (**d**) 1.1 V, 2.25 g of Fe_2_O_3_ + 0.75 g of ZnO (Exp. no. 1-1); (**e**) 1.1 V, 1.50 g of Fe_2_O_3_ + 1.50 g of ZnO (Exp. no. 1-2); and (**f**) 1.1 V, 0.75 g of Fe_2_O_3_ + 2.25 g of ZnO (Exp. no. 1-3).

Figure 10 shows the SEM-EDS results of the cross-section of the cathode surface before and after electrolysis with varying Fe_2_O_3_ to ZnO ratios from 3:1 to 1:3. Compared to the smooth surface observed in the electrode before electrolysis shown in Figure 10a, the electrode recovered after electrolysis in Figure 10b,c showed a rough surface owing to the dendritic growth of the reduced Fe. In addition, several of these coarse particles were partially detached from the electrode surface. Such morphologies have been reported for the reduction of solid-state metal oxides on solid electrodes, where the deposits tend to exhibit weak adhesion to the solid substrate because of their branched structure and limited interfacial contact [38].

As shown in Figure 10d–f, regardless of the composition ratio of the Fe_2_O_3_ and ZnO feedstock mixtures, only metallic Fe was observed on the surface of the Fe cathode, while Zn, B, and Na were not observed. Although the mean cathodic current during electrolysis decreased from 10.37 mA to 1.89 mA as the Fe_2_O_3_ concentration in the molten oxides decreased (see Figure S2a in the Supplementary Materials), the selective reduction of Fe oxide proceeded. The selective reduction of Fe oxide over ZnO was achieved via electrolysis in B_2_O_3_–Na_2_O molten oxides at 1173 K when the ratio of Fe_2_O_3_ to ZnO ranged from 3:1 to 1:3. Therefore, EAF dust with various concentrations of Fe oxide and ZnO can be utilized as feedstock to produce Fe metal using our developed molten oxide system.

#### 4.2.2. Reduction of ZnO from B_2_O_3_–Na_2_O–ZnO Melt

Before conducting the co-reduction of Fe oxide and ZnO, the reduction behavior of ZnO during electrolysis in B_2_O_3_–Na_2_O molten oxides at 1173 K was investigated. Electrolysis was conducted for 1–3 h using an Fe cathode and a Pt anode by applying a cell voltage of 1.6 V. The applied cell voltage was determined by considering the results of the CV measurements shown in Figure 9, which indicate that ZnO can be reduced at 1.21 V, while the decomposition of the supporting electrolyte occurs at a higher cell voltage of 1.65 V.

**Figure 11:**
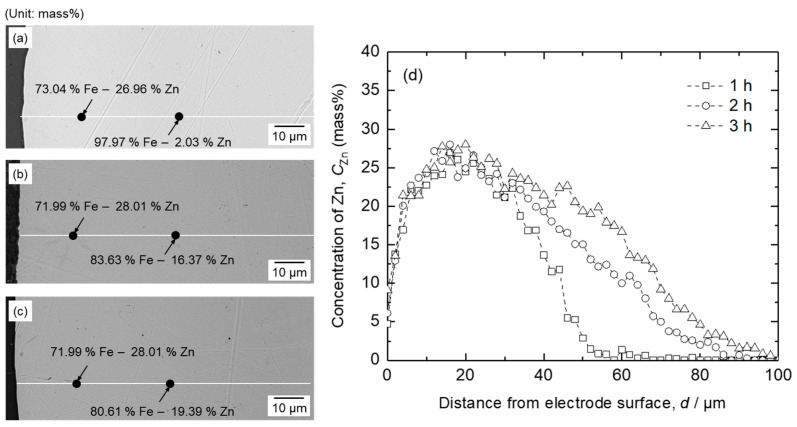
SEM-EDS results for the cathode following the electrolysis of B_2_O_3_–Na_2_O–ZnO at 1173 K by applying a cell voltage of 1.6 V for (**a**) 1 h, (**b**) 2 h, and (**c**) 3 h. (**d**) EDS line result of Zn concentration in the Fe cathode as a function of distance from the electrode surface.

Figure 11 shows the SEM-EDS results for the cathode recovered after electrolysis. As shown in Figure 11a–c, an Fe–Zn solid solution was observed at the cathode, indicating the reduction of ZnO during electrolysis. The electrode exhibited a smooth surface compared with that of the electrode surface with only electrodeposited Fe metal. The Zn concentration exhibited its highest value of 26.96–28.01 mass% in the Fe cathode at 16–20 μm from the surface of the cathode, which was close to its maximum solubility. As shown in Figure 11d, at depths between 18 μm and 100 μm from the cathode surface, Zn diffused into the interior of the Fe cathode. This resulted in a gradual decrease in Zn concentration from its maximum concentration to below the detection limit. In addition, Figure 11d shows that the Zn concentration decreases more gradually with increasing electrolysis duration because a larger amount of Zn is reduced.

Figure 11d shows that at depths of 0–18 μm from the cathode, the Zn concentration increased from the surface of the cathode to its highest value. The Zn concentration at the surface of the cathode was only 4.71–9.12 mass%, likely due to the volatilization of Zn. As shown in Figure 8, Zn exhibited a high vapor pressure of 10^−2^ atm, even when the concentration of Zn in the Fe–Zn alloy was 0.2 mass% at 1173 K. Therefore, Zn at the surface of the Fe cathode can evaporate during electrolysis, resulting in a low concentration.

#### 4.2.3. Co-Reduction of Fe Oxide and ZnO from B_2_O_3_–Na_2_O–Fe_2_O_3_–ZnO Melt

Although the selective reduction of Fe oxides from the mixture of Fe_2_O_3_ and ZnO via electrolysis at 1.1 V was confirmed, a low cathodic current was observed during electrolysis. Therefore, electrolysis of a mixture of Fe_2_O_3_ and ZnO was conducted by applying 1.6 V to investigate the reduction behavior of the oxides while increasing the cathodic current. Electrolysis was conducted in B_2_O_3_–Na_2_O–Fe_2_O_3_–ZnO molten oxides using feedstocks with different ratios of Fe_2_O_3_ to ZnO in Table 2, for 1 h using an Fe cathode and a Pt anode at 1173 K. When electrolysis was conducted at 1.6 V, the co-reduction of Fe and Zn oxides to produce the Fe–Zn alloy was expected considering the decomposition voltages of Fe oxide and ZnO, as shown in Figure 9. However, Zn can be separated from the Fe–Zn alloy through vacuum distillation owing to the large vapor pressure difference between Fe and Zn.

**Figure 12:**
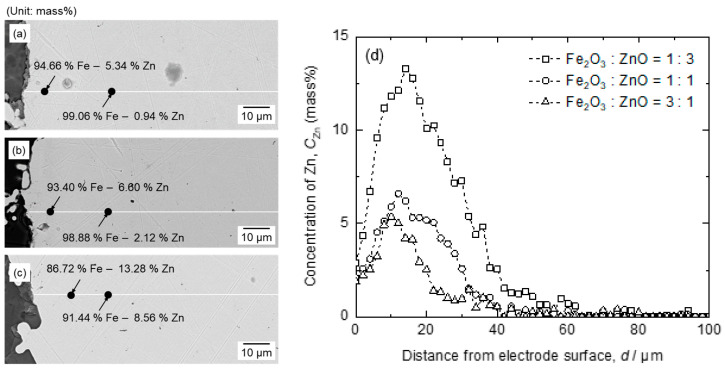
SEM-EDS results for the cathode following the electrolysis of B_2_O_3_–Na_2_O–Fe_2_O_3_–ZnO at 1173 K for 1 h by applying a cell voltage of 1.6 V in following feedstocks: (**a**) 2.25 g of Fe_2_O_3_ + 0.75 g of ZnO (Exp. no. 2-1); (**b**) 1.50 g of Fe_2_O_3_ + 1.50 g of ZnO (Exp. no. 2-2); (**c**) 0.75 g of Fe_2_O_3_ + 2.25 g of ZnO (Exp. no. 2-3). (**d**) EDS line analysis result of Zn concentration in Fe cathode as a function of distance from the electrode surface.

Figure 12 shows the SEM-EDS results for the cross-section of the cathode recovered after electrolysis conducted for 1 h at 1.6 V, using Fe_2_O_3_ and ZnO feedstocks at various ratios. As shown in Figure 12, an Fe–Zn solid solution was observed at the Fe cathode, indicating that ZnO was reduced on the Fe cathode. The Zn concentration in the Fe cathode increased as the ZnO concentration in the feedstock increased, exhibiting its highest value of 5.34–13.28 mass% Zn, at 10–14 μm from the surface of the cathode. The Zn concentration gradually decreased after reaching its highest value owing to its diffusion into the interior of the Fe cathode. Meanwhile, the concentration of Zn near the surface of the Fe cathode was 1.89–2.87 mass%, owing to the evaporation of Zn at the surface of the cathode.

As shown in Figure 12a–c, the electrode exhibited a rough surface owing to the dendritic growth of reduced Fe. The dendritic Fe deposit contained 6.98–17.87 mass% Zn (see Figure S3 in the Supplementary Materials). Therefore, the co-reduction of Fe oxide and ZnO occurred when electrolysis was conducted at a cell voltage of 1.6 V.

**Figure 13:**
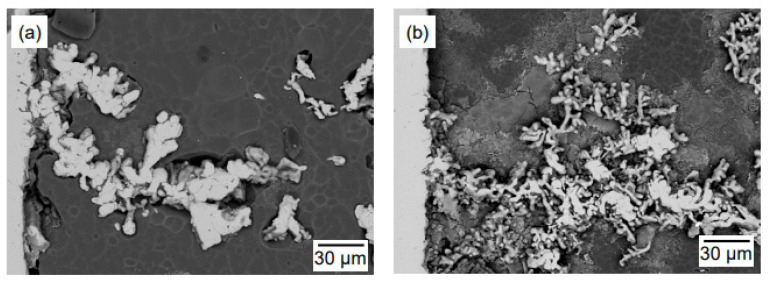
SEM images of the cathode surface after electrolysis at 1173 K for 5 h under the following conditions: (**a**) 1.1 V, 0.75 g of Fe_2_O_3_ + 2.25 g of ZnO; (**b**) 1.6 V, 0.75 g of Fe_2_O_3_ + 2.25 g of ZnO.

The morphology of the dendritic Fe deposit was further analyzed to evaluate the effect of cell voltage. In Figure 10b, when the applied cell voltage was 1.1 V, the reduced Fe appeared as coarse and compact deposits localized near the cathode surface. In contrast, at 1.6 V, the deposits exhibited a finer dendritic morphology and were more widely distributed in the electrolyte. Figure 13 shows the SEM images of the cathode after electrolysis for 5 h under the same experimental condition as in Exp. nos. 1-3 and 2-3. As the electrolysis time was extended to 5 h, the morphological features observed at 1 h became more pronounced, with coarser dendritic deposits at 1.1 V and finer, more branched dendritic deposits at 1.6 V. Thus, applying a higher cell voltage of 1.6 V promoted a higher nucleation rate and finer dendrite growth.

The electrolysis results indicate that the co-reduction of Fe oxide and ZnO is attainable at 1173 K with an applied cell voltage of 1.6 V, when the weight ratio of Fe_2_O_3_ to ZnO in the feedstock ranges from 3:1 to 1:3. Fe is reduced on the surface of the Fe cathode and Zn is reduced at the Fe cathode and on the Fe deposit. In addition, a mean cathodic current of 55.15–30.92 mA was observed during electrolysis at an applied cell voltage of 1.6 V. This value is significantly higher than that observed during electrolysis at 1.1 V (see Figure S2 in the Supplementary Materials), indicating an enhanced overall reduction rate of oxides.

Throughout the electrolysis experiments, the stability of the supporting electrolyte was maintained owing to its low vapor pressure and by controlling the cell voltage below the decomposition voltage of B_2_O_3_. Regarding electrode stability, Pt is thermodynamically stable even under high $p_{O_{2}}$ conditions. Furthermore, an SEM image after electrolysis revealed no detectable degradation of the anode surface and the thickness remained unchanged, confirming its stability (see Figure S4 in the Supplementary Materials).

### 4.3. Vacuum Distillation of the Fe–Zn Alloys

To investigate the feasibility of separating high-purity Fe and Zn from the Fe–Zn alloy, vacuum distillation of the Fe–27.1 mass% Zn alloys was conducted at 1000–1200 K for 1–12 h. The concentration of Zn in the Fe–Zn alloy was selected by considering its maximum concentration in the Fe–Zn alloys obtained after electrolysis. Owing to the large difference in vapor pressure between Fe and Zn, vacuum distillation at high temperatures is an effective method for separating Fe and Zn from their alloys (see Figure S5 in the Supplementary Materials).

**Figure 14:**
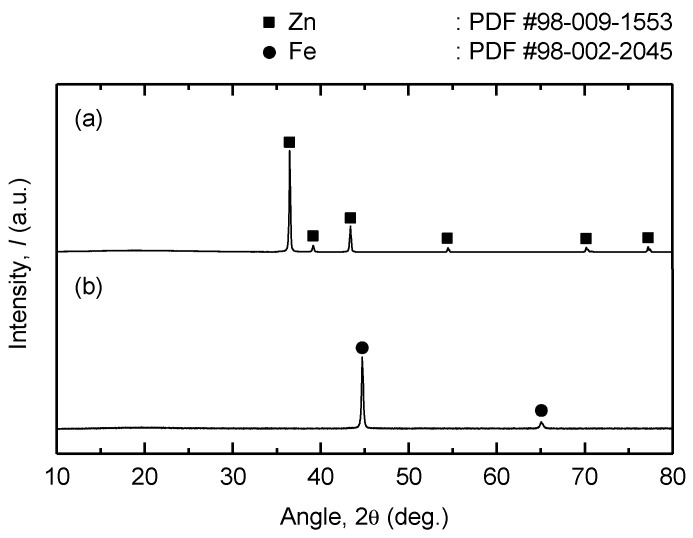
XRD results for the (**a**) deposit obtained from the low-temperature part of the reactor and (**b**) residue at the bottom of the reactor following the vacuum distillation of the Fe–Zn alloy at 1200 K for 12 h.

Figure 14 shows the XRD results of the deposit obtained from the low-temperature part of the reactor and the residue at the bottom of the reactor following vacuum distillation of the Fe–Zn alloy at 1200 K for 12 h. Table 4 shows the ICP-OES results of the Fe–Zn alloy before distillation, Zn deposit after distillation at 1200 K for 12 h, and residues after distillation at 1000–1200 K for 1–12 h. The results show that Zn was separated from the Fe–Zn alloy and condensed at the low-temperature part of the reactor with a purity of ≥99.996%. In addition, the residue at the bottom of the reactor was Fe with a purity of ≥99.9% (see Figure S6 in the Supplementary Materials for detailed information of vacuum distillation). Thus, vacuum distillation enabled the separation and recovery of high-purity Zn and Fe from Fe–Zn alloys.

The effect of distillation time and temperature on the recovery efficiency of Zn was evaluated based on the ICP results in Table 4. The Zn recovery efficiency was calculated using Equation (6), where *w*_residue_ is the weight of the residue at the bottom of the reactor, *w*_feed_ is the weight of the Fe–Zn alloy before vacuum distillation, *C*_Zn_feed_ is the concentration of Zn in the Fe–Zn alloy before vacuum distillation, and *C*_Zn_residue_ is the concentration of Zn in the residue at the bottom of the reactor.

*R* (%) = 100 × {1 − (*w*_residue_/*w*_feed_)/(*C*_Zn_feed_/*C*_Zn_residue_)} (6)

**Figure 15:**
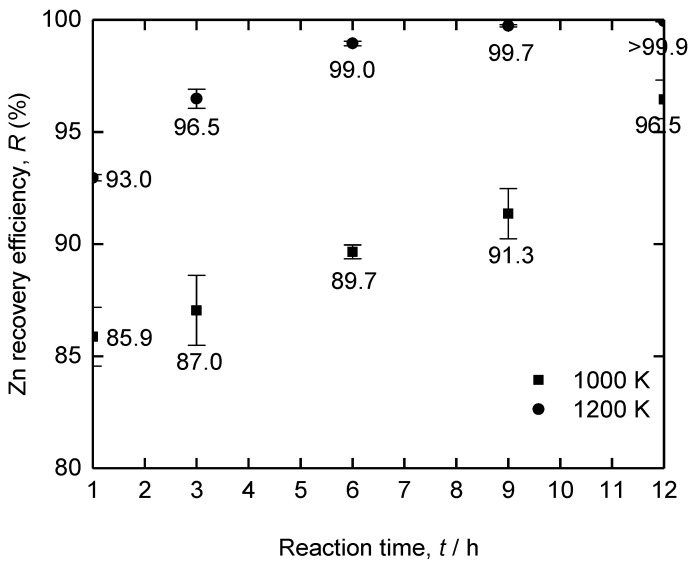
Efficiency of Zn recovery following vacuum distillation of the Fe–Zn alloy at 1000 K and 1200 K for 1–12 h.

Figure 15 shows the Zn recovery efficiency obtained after vacuum distillation of the Fe–Zn alloy at 1000 K and 1200 K for 1–12 h. At 1000 K, the Zn recovery efficiency increased from 85.9% at 1 h to 96.5% at 12 h. However, the Zn in Fe–Zn alloys was not completely recovered even when distillation was conducted for 12 h. This is because the vapor pressure of Zn was not sufficient for volatilization, as it decreases below 10^−2^ atm when its concentration in the Fe–Zn alloy decreases to 1.2 mass% during vacuum distillation.

Meanwhile, at 1200 K, the efficiency increased from 93.0% to 99.0% as the distillation time increased from 1 h to 6 h and further increased to 99.7–99.9% after 9–12 h. The increased recovery efficiency of Zn from the Fe–Zn alloy at 1200 K compared to 1000 K is due to the increase in vapor pressure of Zn, as shown in Figure 8. Thus, a sufficient amount of Zn was recovered from the Fe–Zn alloy via vacuum distillation at 1200 K for 6 h with a recovery efficiency of ≥99%.

## 5. Conclusions

The electrochemical behavior and electrolysis of Fe_2_O_3_ and ZnO in a B_2_O_3_–Na_2_O molten oxide electrolyte at 1173 K were investigated to develop a CO_2_-free process for the recovery of Fe and Zn from EAF dust. The CV measurement results revealed that the decomposition voltages of FeO, ZnO, and B_2_O_3_ in the electrolyte were 0.97 V, 1.21 V, and 1.65 V, respectively, as expected from thermodynamic calculations. When electrolysis was conducted at a cell voltage of 1.1 V for 1 h using an Fe cathode and a Pt anode, the selective reduction of Fe oxide was observed, resulting in the formation of coarse metallic deposits on the cathode surface. At an applied cell voltage of 1.6 V for 1 h, an Fe–Zn alloy was produced owing to the co-reduction of Fe oxide and ZnO, and the maximum concentration of Zn in the Fe cathode increased from 5.34 mass% to 13.28 mass% when the ratio of ZnO in the feedstock increased from 0.25 to 0.75, respectively. When vacuum distillation was conducted at 1200 K for 12 h, Zn metal with a purity of ≥99.996% was obtained, with a recovery efficiency of ≥99.9%.

## Figures and Tables

**Table 1:** Previous studies on the electrolysis of Fe oxide feedstocks in various electrolytes.

Method	Electrolyte	Feedstock		Temp., *T*/K	Electrode for Electrolysis		Cathode Product	Cell Voltage, *E*/V	Faradaic Efficiency, (%)	Ref.
		Type	FeO_x_ Conc., (mass%)		Cathode	Anode				
Molten oxide electrolysis	Al_2_O_3_–CaO–MgO	Fe_3_O_4_	10	1838	Mo	Cr_90_Fe_10_	Fe	3.8	34	[8]
	Al_2_O_3_–CaO–MgO–SiO_2_	Fe_2_O_3_	10	1873	Mo	Graphite	Fe	2	32	[9]
	Al_2_O_3_–MgO–SiO_2_	Fe_3_O_4_	15	1823	Pt–Rh	Pt	Fe	3	36	[10]
	Al_2_O_3_–CaO–SiO_2_	Fe_2_O_3_	5	1773–1873	Mo	Graphite	Fe	–	64–83	[11]
	Al_2_O_3_–CaO–MgO–SiO_2_	Fe_2_O_3_	9.1	1823	Mo	Ir	Fe	2	25 ^a^	[12]
	Al_2_O_3_–CaO–MgO–SiO_2_	Fe_2_O_3_	5	1848	Mo	Ir	Fe	–	–	[13]
	B_2_O_3_–Na_2_O	Fe_2_O_3_	5	1273	Pt	Pt	Fe	1.4	33.2	[14]
	K_2_MoO_4_–Fe_2_O_3_	Fe_2_O_3_	–	1273	Steel	Steel	Fe–Mo	–	70.77	[15]
	Al_2_O_3_–CaO–MgO–SiO_2_	Fe_2_O_3_, NiO	15	1723	W	Graphite	Fe–Ni	–	46	[16]
Molten salt electrolysis	CaCl_2_–KF	Fe_2_O_3_	1.7	1100	Fe	Fe_3_O_4_	Fe	–	–	[17]
	CaCl_2_–CaF_2_	Fe_2_O_3_	1.5	1163	Fe	Fe_3_O_4_	Fe	–	92	[18]
	NaCl–CaCl_2_	Fe_2_O_3_ ^b^	–	1073	Fe_2_O_3_	Graphite	Fe	1.2	95.3	[19]
	LiCl	Fe_2_O_3_ ^b^	–	933	Fe_2_O_3_	Graphite	Fe	0.97	97	[20]
	CaCl_2_	Fe_2_O_3_ ^b^	–	1073	Fe_2_O_3_	Graphite	Fe	1.8	90	[21]
	NaCl–CaCl_2_	ZnFe_2_O_4_ ^b^	–	1073	ZnFe_2_O_4_	Graphite	Fe ^c^	1.8	–	[22]
	NaCl–KCl	Fe_2_O_3_, Al_2_O_3_ ^b^	–	1123	Fe_2_O_3_– Al_2_O_3_	Graphite	FeO–Al_2_O_3_	2.3	–	[23]
	CaCl_2_	Fe_2_O_3_, TiO_2_ ^b^	–	1223	Fe_2_O_3_– TiO_2_	Graphite	Fe–Ti	3	–	[24]
	CaCl_2_	Fe_2_O_3_, Tb_4_O_7_^b^	–	1173	Fe_2_O_3_– Tb_4_O_7_	Graphite	Fe–Tb	2.6	97	[25]
Molten carbonate electrolysis	Na_2_CO_3_–K_2_CO_3_	Fe_2_O_3_ ^b^	–	1023	Fe_2_O_3_	Ni–Cu–Fe	Fe	2	93.6	[26]
Molten hydroxide electrolysis	NaOH	Fe_2_O_3_ ^b^	–	803	Fe_2_O_3_	Ni	Fe	1.7	89	[27]
	NaOH	Fe_2_O_3_ ^b^	–	773	Fe_2_O_3_	Ni–Si–Al	Fe	1.7	30	[28]

^a^ anodic current efficiency. ^b^ direct solid-state electrolysis using oxide cathode as feedstock. ^c^ Zn volatilized.

**Table 2:** Experimental conditions for the electrolysis of Fe_2_O_3_ and/or ZnO using an Fe cathode and Pt anode at 1173 K.

Exp. No. ^a^	Weight of Feed, *w*_feed_/g		Mass Ratio of Oxide to Feed, *r*_oxide/feed_		Applied Cell Voltage, *E*/V	Time, *t*/h
	Fe_2_O_3_	ZnO	Fe_2_O_3_	ZnO		
1-1	2.25	0.75	0.75	0.25	1.10	1
1-2	1.50	1.50	0.50	0.50	1.10	1
1-3	0.75	2.25	0.25	0.75	1.10	1
2-1	2.25	0.75	0.75	0.25	1.60	1
2-2	1.50	1.50	0.50	0.50	1.60	1
2-3	0.75	2.25	0.25	0.75	1.60	1
3-1	0.00	3.00	0.00	1.00	1.60	1
3-2	0.00	3.00	0.00	1.00	1.60	2
3-3	0.00	3.00	0.00	1.00	1.60	3

^a^ Experimental conditions: Weight of supporting electrolyte = 97 g; 73 mass% B_2_O_3_–27 mass% Na_2_O mixture.

**Table 3:** Theoretical standard decomposition voltages of selected oxides at 1173 K, 1273 K and 1373 K.

Reaction	Decomposition Voltage, *E*/V			Phase Transformation
	1173 K	1273 K	1373 K	
FeO (*s*) = Fe (*s*) + 1/2 O_2_ (*g*)	0.98	0.94	0.91	Fe (BCC) → Fe (FCC) at 1184.81 K
ZnO (*s*) = Zn (*l,g*) + 1/2 O_2_ (*g*)	1.19	1.09	0.98	Zn (*l*) → Zn (*g*) at 1181.47 K
B_2_O_3_ (*l*) = 2 B (*s*) + 3/2 O_2_ (*g*)	1.70	1.66	1.62	–
Na_2_O (*s*) = 2 Na (*g*) + 1/2 O_2_ (*g*)	1.33	1.18	1.03	Na_2_O (*β*) → Na_2_O (*α*) at 1243 K
Fe_2_O_3_ (*s*) = 2 Fe (*s*) + 3/2 O_2_ (*g*)	0.90	0.86	0.81	Fe (BCC) → Fe (FCC) at 1184.81 K
Fe_3_O_4_ (*s*) = 3 Fe (*s*) + 2 O_2_ (*g*)	0.96	0.92	0.88	Fe (BCC) → Fe (FCC) at 1184.81 K
Fe_2_O_3_ (*s*) = 2 FeO (*s*) + 1/2 O_2_ (*g*)	0.74	0.68	0.62	–

**Table 4:** ICP-OES results of Fe–Zn alloy before distillation, Zn deposit after distillation at 1200 K for 12 h, and residues after distillation at 1000–1200 K for 1–12 h.

Sample	Temp., *T*/K	Time, *t*/h	Concentration of Element *i*, *C_i_* (mass%)	
			Fe	Zn
Fe–Zn feed	–	–	72.894	27.105
Zn deposit	1200	12	0.003	99.996
Residues of No. 1	1000	1	94.955	5.044
Residues of No. 2	1000	3	95.380	4.619
Residues of No. 3	1000	6	96.336	3.663
Residues of No. 4	1000	9	96.764	3.235
Residues of No. 5	1000	12	98.667	1.332
Residues of No. 6	1200	1	97.407	2.592
Residues of No. 7	1200	3	98.703	1.296
Residues of No. 8	1200	6	99.610	0.389
Residues of No. 9	1200	9	99.901	0.098
Residues of No. 10	1200	12	99.978	0.021

## Data Availability

The original contributions presented in the study are included in the article. Further inquiries can be directed to the corresponding author(s).

## Supplementary Materials

The following supporting information can be downloaded at https://www.mdpi.com/article/10.3390/ma18174116/s1, Figure S1: Photograph of experimental apparatus used for cyclic voltammetry measurement and electrolysis in this study; Figure S2: Current during electrolysis of Fe_2_O_3_ and ZnO mixture in 73 mass% B_2_O_3_–Na_2_O molten oxide electrolyte at 1173 K for 1 h, by applying a cell voltage of (a) 1.1 V and (b) 1.6 V; Figure S3: SEM-EDS results for the cathode after electrolysis of B_2_O_3_–Na_2_O–Fe_2_O_3_–ZnO at 1173 K for 1 h by applying a cell voltage of 1.6 V in following feedstocks; (a) 2.25 g of Fe_2_O_3_ + 0.75 g of ZnO (Exp. no. 2-1); (b) 0.75 g of Fe_2_O_3_ + 2.25 g of ZnO (Exp. no. 2-3); Figure S4: SEM images of the Pt anode (a) before and (b) after electrolysis; Figure S5: Vapor pressure of Fe and Zn at elevated temperatures; Figure S6: Temperature profile of the reactor at 1200 K and photographs of deposit and residue obtained after vacuum distillation of Fe–Zn alloy at 1200 K for 12 h.

## References

[1] World Steel Association World Steel in Figures 2025. https://worldsteel.org/data/world-steel-in-figures/world-steel-in-figures-2025/.

[2] US Geological Survey Mineral Commodity Summaries 2025: Iron and Steel. https://pubs.usgs.gov/periodicals/mcs2025/mcs2025-iron-steel.pdf.

[3] Sohn H. (2020). Recycling of Common Metals.

[4] World Steel Association Steel’s Contribution to a Low Carbon Future and Climate Resilient Societies. https://www.acero.org.ar/wp-content/uploads/2020/02/Position_paper_climate_2020_vfinal.pdf.

[5] Pistorius P.C. Energy requirements for electrification of ironmaking and steelmaking. Proceedings of the Southern African Pyrometallurgy, Misty Hills Conference Centre.

[6] Patisson F., Mirgaux O. (2020). Hydrogen ironmaking: How it works. Metals.

[7] IEAGHG (2024). Clean Steel: An Environmental and Technoeconomic Outlook of a Disruptive Technology. IEAGHG Technical Report.

[8] Allanore A., Yin L., Sadoway D.R. (2013). A new anode material for oxygen evolution in molten oxide electrolysis. Nature.

[9] Zhang K., Jiao H., Zhou Z., Jiao S., Zhu H. (2016). Electrochemical behavior of Fe (III) ion in CaO–MgO–SiO_2_–Al_2_O_3_–NaF–Fe_2_O_3_ melts at 1673 K. J. Electrochem. Soc..

[10] Wiencke J., Lavelaine H., Panteix P.-J., Petitjean C., Rapin C. (2018). Electrolysis of iron in a molten oxide electrolyte. J. Appl. Electrochem..

[11] Liu J., Zhang Y. (2023). Electroreduction mechanism and electrodeposition of ferric ions in CaO–SiO_2_–Al_2_O_3_–Fe_2_O_3_ slags at 1773 K. Can. Metall. Q..

[12] Kim H., Paramore J.D., Allanore A., Sadoway D.R. (2010). Stability of iridium anode in molten oxide electrolysis for ironmaking: Influence of slag basicity. ECS Trans..

[13] Wang D., Gmitter A.J., Sadoway D.R. (2011). Production of oxygen gas and liquid metal by electrochemical decomposition of molten iron oxide. J. Electrochem. Soc..

[14] Choi H.-G., Choi S., Kim M.-K., Jang J., Nam K.T., Jung I.-H., Yi K.-W. (2021). Electrolysis of iron with oxygen gas evolution from molten sodium borate electrolytes. Ironmak. Steelmak..

[15] Park K.W., Sohn I. (2023). Electrochemical reduction of K_2_MoO_4_–Fe_2_O_3_ binary melts using a consumable steel electrode to produce ferromolybdenum alloys. J. Sustain. Metall..

[16] Zhou Z., Jiao H., Tu J., Zhu J., Jiao S. (2017). Direct production of Fe and Fe–Ni alloy via molten oxides electrolysis. J. Electrochem. Soc..

[17] Haarberg G.M., Kvalheim E. (2017). Electrochemical behavior of dissolved Fe_2_O_3_ in molten CaCl_2_–KF. J. Electrochem. Soc..

[18] Haarberg G.M., Kvalheim E., Rolseth S., Murakami T., Pietrzyk S., Wang S. (2007). Electrodeposition of iron from molten mixed chloride/fluoride electrolytes. ECS Trans..

[19] Li H., Jia L., Liang J.-l., Yan H.-y., Cai Z.-y., Reddy R.G. (2019). Study on the direct electrochemical reduction of Fe_2_O_3_ in NaCl–CaCl_2_ melt. Int. J. Electrochem. Sci..

[20] Xie K., Kamali A.R. (2019). Molten salt electrochemical production and in situ utilization of hydrogen for iron production. Int. J. Hydrogen Energy.

[21] Li G., Wang D., Chen Z. (2009). Direct reduction of solid Fe_2_O_3_ in molten CaCl_2_ by potentially green process. J. Mater. Sci. Technol..

[22] Liu C., Liang J., Li H., Yan H., Zheng S., Cao W., Wang L. (2021). The electrochemical reduction mechanism of ZnFe_2_O_4_ in NaCl–CaCl_2_ melts. Crystals.

[23] Xu Y., Yan H., Jing Z., Qi X., Li H., Liang J. (2021). Effect of Fe_2_O_3_ on electro-deoxidation in Fe_2_O_3_–Al_2_O_3_–NaCl–KCl system. Crystals.

[24] Panigrahi M., Iizuka A., Shibata E., Nakamura T. (2012). Fe–Ti alloy production by electrolytic reduction of (Fe, Ti) oxide electrodes in molten calcium chloride. TT Chen Honorary Symposium on Hydrometallurgy, Electrometallurgy and Materials Characterization.

[25] Qiu G., Wang D., Ma M., Jin X., Chen G.Z. (2006). Electrolytic synthesis of TbFe_2_ from Tb_4_O_7_ and Fe_2_O_3_ powders in molten CaCl_2_. J. Electroanal. Chem..

[26] Tang D., Yin H., Xiao W., Zhu H., Mao X., Wang D. (2013). Reduction mechanism and carbon content investigation for electrolytic production of iron from solid Fe_2_O_3_ in molten K_2_CO_3_–Na_2_CO_3_ using an inert anode. J. Electroanal. Chem..

[27] Cox A., Fray D.J. (2008). Mechanistic investigation into the electrolytic formation of iron from iron (III) oxide in molten sodium hydroxide. J. Appl. Electrochem..

[28] Wang S., Ge J., Hu Y., Zhu H., Jiao S. (2013). Electrochemical reduction of iron oxide in molten sodium hydroxide based on a Ni_0_._94_Si _0_._04_Al_0.02_ metallic inert anode. Electrochim. Acta.

[29] Kim M.-K., Jung I.-H. (2022). Coupled experimental phase diagram study and thermodynamic optimization of the Na_2_O–B_2_O_3_–Fe_2_O_3_ system in air. Calphad.

[30] Kim M.-K., Jung I.-H. (2024). Coupled phase diagram study and thermodynamic modeling of the Na_2_O–B_2_O_3_–ZnO system. J. Eur. Ceram. Soc..

[31] Claes P., Coq J., Glibert J. (1988). Electrical conductivity of molten B_2_O_3_–Na_2_O mixtures. Electrochim. Acta.

[32] Al-Zaibani M., Althobiti R., El Agammy E., Alzahrani E., El-Damrawi G., Doweidar H., Al-Muntaser A. (2025). Electrical conduction in ternary Na_2_O–ZnO–B_2_O_3_ glasses; a unique dependence on the mobility of Na^+^ ions as main charge carriers. J. Non-Crystal. Solids.

[33] Tijaria M., Sharma Y., Kumar V., Dahiya S., Dalal J. (2021). Effect of Na_2_O on physical, structural and electrical properties of borate glasses. Mater. Today Proc..

[34] Kim H., Paramore J., Allanore A., Sadoway D.R. (2011). Electrolysis of molten iron oxide with an iridium anode: The role of electrolyte basicity. J. Electrochem. Soc..

[35] Asai K., Yokokawa T. (1982). Thermodynamic activity of Na_2_O in Na_2_O–B_2_O_3_–SiO_2_ melt. Trans. Jpn Inst. Met..

[36] Konakov V. (2002). A study of the acid-base properties of melts in the Na_2_O–B_2_O_3_–SiO_2_ system: II. Composition Joins with Sodium Oxide Contents of 25, 30, and 35 mol%. Glass Phys. Chem..

[37] Park J.H., Min D.J. (2001). Thermodynamic behavior of Na_2_O–B_2_O_3_ melt. Metall. Mater. Trans. B.

[38] Popov K.I., Nikolić N.D. (2012). General theory of disperse metal electrodeposits formation. Electrochemical Production of Metal Powders.

