# Supplementary material for: Investigation of Low-Temperature Molten Oxide Electrolysis of a Mixture of Hematite and Zinc Oxide
Source: Materials (Basel). 2025 Sep 2;18(17):4116. doi: 10.3390/ma18174116 (PMC12429705; doi:10.3390/ma18174116)
Supplement: Supplementary file 1 [file materials-18-04116-s001.zip › materials-3816593-supplementary.pdf]

materials

Supplementary material

Investigation of Low-Temperature Molten Oxide Electrolysis of a Mixture of Hematite and Zinc Oxide

Joongseok Kim [1], In-Ho Jung [1,2], Jungshin Kang [3,4,*] and Kyung-Woo Yi [1,2,*]

[1] Department of Materials Science and Engineering, Seoul National University, 1 Gwanak-ro, Gwanak-gu, Seoul 08826, Republic of Korea; joongseok10@snu.ac.kr (J.K.); in-ho.jung@snu.ac.kr (I.-H.J.)
[2] Research Institute of Advanced Materials, Seoul National University, 1 Gwanak-ro, Gwanak-gu, Seoul 08826, Republic of Korea
[3] Department of Energy Resources Engineering, Seoul National University, 1 Gwanak-ro, Gwanak-gu, Seoul 08826, Republic of Korea
[4] Research Institute of Energy and Resources, Seoul National University, 1 Gwanak-ro, Gwanak-gu, Seoul 08826, Republic of Korea
* Correspondence: kangjs@snu.ac.kr (J.K.); yikw@snu.ac.kr (K.-W.Y.); Tel.: +82-2-880-8715 (J.K.); +82-2-880-8307 (K.-W.Y.)

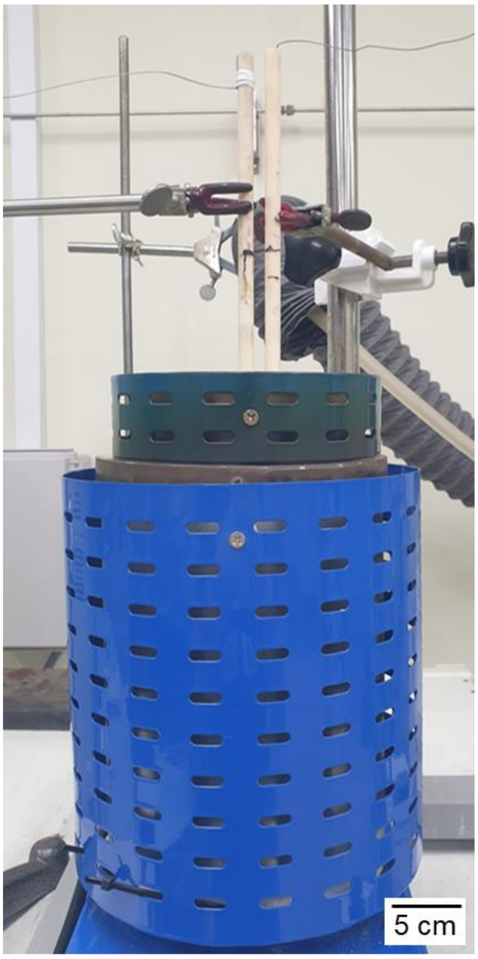

Figure S1. Photograph of experimental apparatus used for cyclic voltammetry measurement and electrolysis in the study.

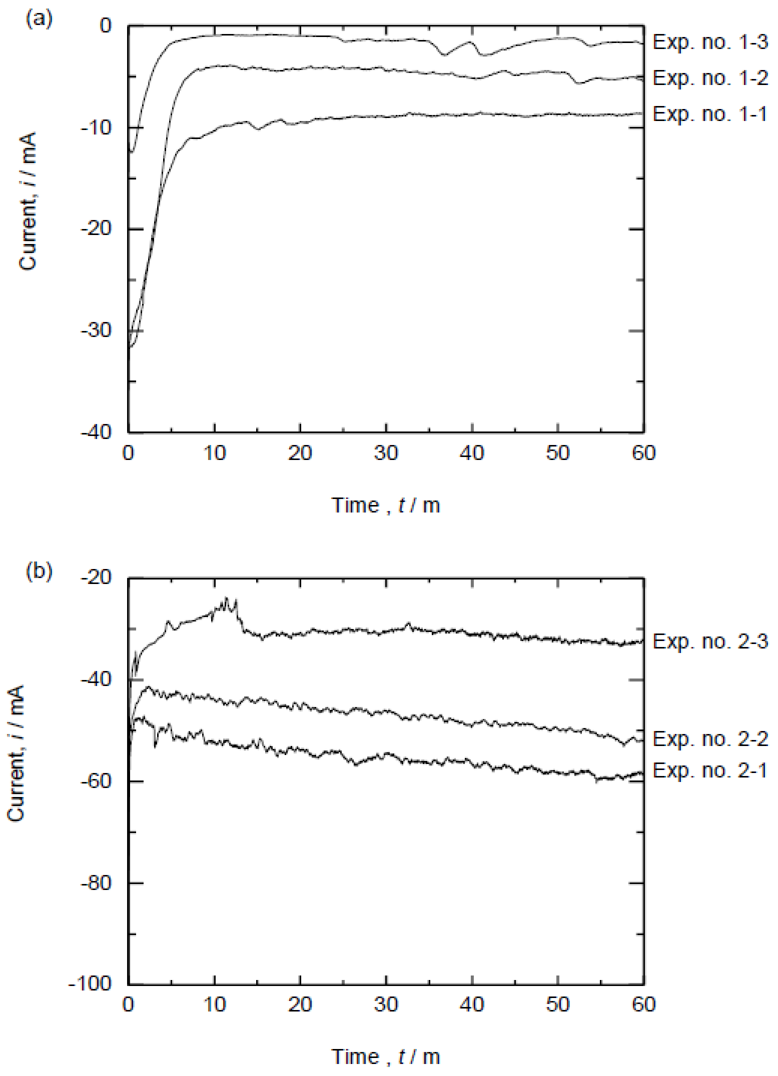

Figure S2. Current density during electrolysis of Fe_2O_3 and ZnO mixture in 73 mass% B_2O_3 – Na_2O molten oxide electrolyte at 1173 K for 1 h, by applying cell voltage of (a) 1.1 V and (b) 1.6 V.

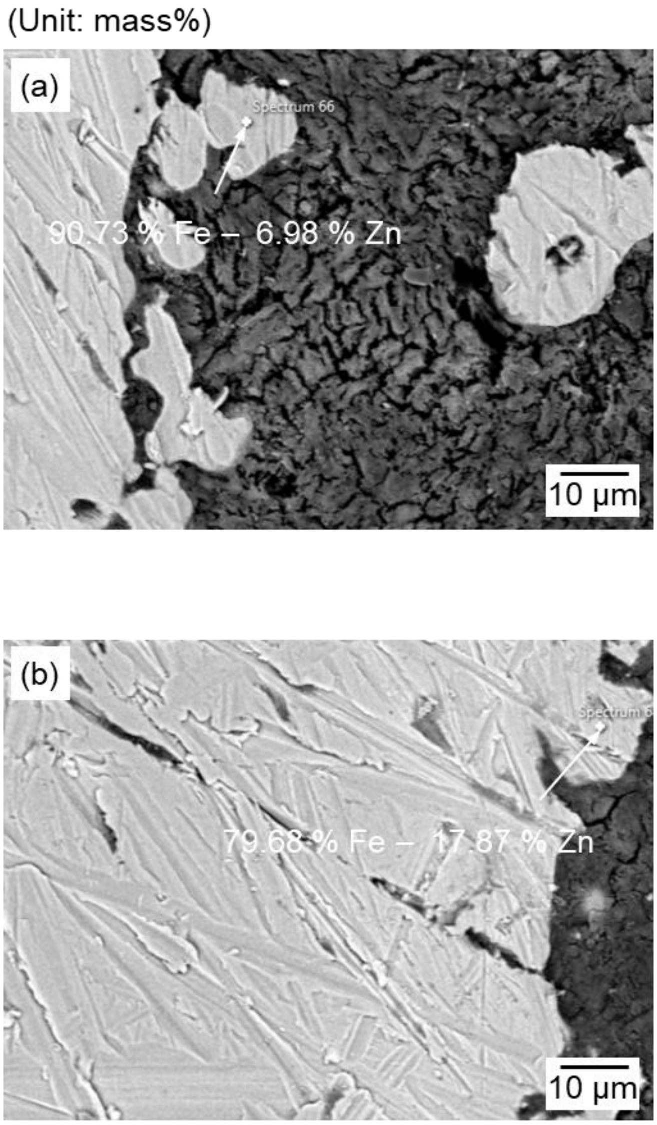

Figure S3. SEM-EDS analysis result of the cathode after electrolysis of B_2O_3 – Na_2O – Fe_2O_3 – ZnO at 1173 K for 1 h by applying cell voltage of 1.6 V in following feedstocks;.**(a)** 2.25 g of Fe_2O_3 + 0.75 g of ZnO (Exp. no. 2-1); (**b**) 0.75 g of Fe_2O_3 + 2.25 g of ZnO (Exp. no. 2-3).

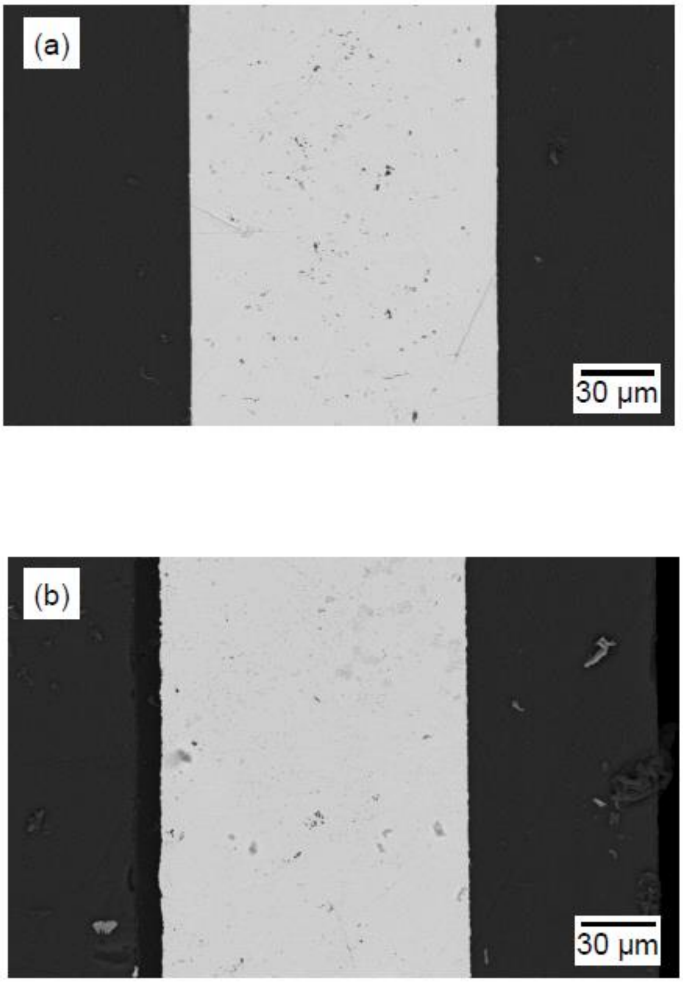

Figure S4. SEM images of the Pt anode (**a**) before and (**b**) after electrolysis.

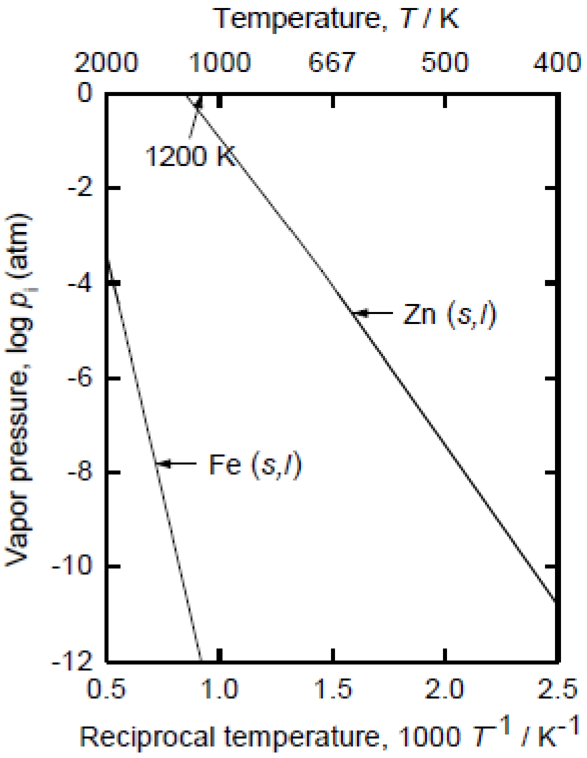

Figure S5. Vapor pressure of Fe and Zn at elevated temperatures.

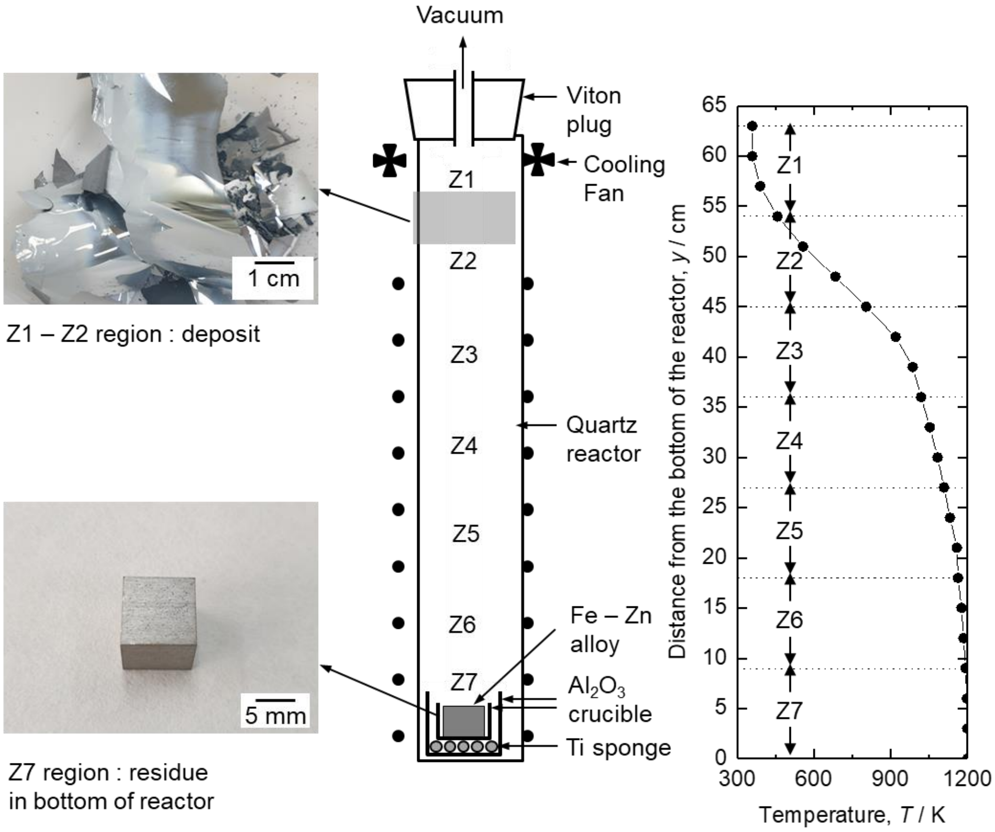

Figure S6. Temperature profile of the reactor at 1200 K and photographs of deposit. and residue obtained after vacuum distillation of Fe – Zn alloy at 1200 K for 12 h
